# Reactive oxygen species and metabolic checkpoints shape plasmacytoid dendritic cell fate in infection and autoimmunity

**DOI:** 10.1016/j.redox.2026.104185

**Published:** 2026-04-22

**Authors:** Lena Rueschpler, Sebastian Schloer

**Affiliations:** a Institute for Immunology, University Medical Centre Hamburg-Eppendorf, Hamburg, Germany; b Research Department Virus Immunology, Leibniz Institute of Virology, Hamburg, Germany

**Keywords:** Plasmacytoid dendritic cells, Interferons, Immunometabolism, Redox signalling, Mitochondria, NAD^+^, Autoimmunity, Viral infection

## Abstract

Plasmacytoid dendritic cells (pDCs) are innate immune sentinels uniquely specialised in the rapid and potent production of type I interferons (IFN–I) during viral infection. While this capacity is essential for antiviral defence, sustained pDC activation is a central feature of numerous autoimmune and inflammatory disorders. Although the molecular pathways governing nucleic acid sensing and IFN-I induction have been extensively characterised, the metabolic and redox mechanisms that support, and limit pDC function remain incompletely understood. Emerging studies reveal that pDC activity is tightly linked to a specialised redox-metabolic programme involving mitochondrial respiration, reactive oxygen species (ROS), and endolysosomal signalling networks. In this review, we integrate current evidence to propose that pDCs operate within a tightly regulated redox window that permits effective acute antiviral responses but renders them vulnerable to metabolic stress and dysregulation upon chronic stimulation. We examine how mitochondrial fitness, NAD^+^ homeostasis, ROS dynamics, and endolysosomal redox control collectively influence pDC activation, resolution of inflammation, and pathogenic persistence. By reframing pDC biology through a redox-metabolic perspective, we highlight new conceptual insights into IFN-I-driven disease and identify potential therapeutic strategies to selectively modulate pathogenic pDC responses.

## Highlights

• A defined redox window enables antiviral IFN-I responses in pDCs.
• ROS act as signalling mediators rather than mere by-products.
• Metabolic checkpoints integrate mitochondrial and lysosomal pathways.
• Chronic stimulation disrupts redox balance and sustains inflammation.
• Targeting redox metabolism may selectively modulate pathogenic pDCs.

## Graphical abstract

**Figure:**
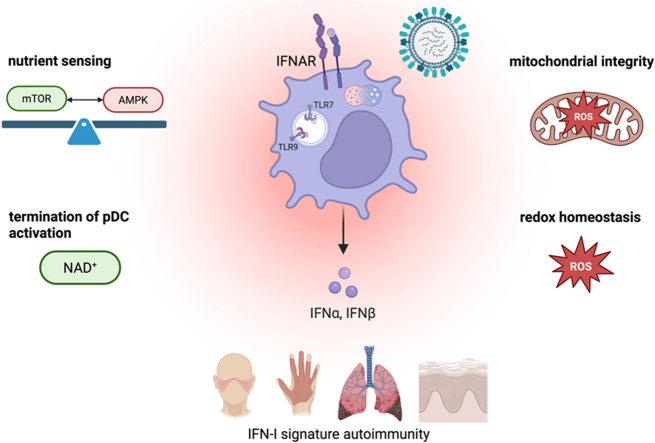

## 1 Introduction

Plasmacytoid dendritic cells (pDCs) are a rare population of innate immune cells, constituting only ∼0.5% of peripheral blood mononuclear cells, that bridge pathogen sensing and systemic antiviral defence. Their defining feature is the ability to produce extraordinarily high levels of IFN-I, particularly IFN-α, within hours of viral encounter [1]^,^ [2]. This capacity is enabled by selective engagement of endosomal Toll-like receptors (TLR7 and TLR9) and constitutive expression of interferon regulatory factor 7 (IRF7), allowing pDCs to respond rapidly to viral nucleic acids without requiring prior priming [3]. While IFN-I production by pDCs is indispensable for early viral control, sustained activation of this pathway is increasingly recognised as pathogenic. Chronic pDC-derived IFN-I signatures are a hallmark of many autoimmune diseases [4] such as systemic lupus erythematosus (SLE) [5], systemic sclerosis (SSc) [6,7], Sjögren's syndrome (SjS) [8], psoriasis [9]^,^ [10] and dermatomyositis (DM) [11]^,^ [12]. In these conditions, pDCs demonstrate tissue infiltration and remain persistently activated by immune complexes containing self-derived nucleic acids, driving an inflammatory feed-forward loop.

The current literature on pDCs has to date predominantly been focused on receptor signalling, transcriptional regulation, and cytokine release. In contrast, the metabolic and redox underpinnings of pDC function have received remarkably little attention. This gap is notable, given that IFN-I production represents one of the most energetically demanding responses in innate immunity and is tightly linked to mitochondrial activity, redox balance, and cellular stress responses [13]^,^ [14]. In other immune cell types, immunometabolism and redox signalling have emerged as central determinants of activation, differentiation, and exhaustion [15]^,^ [16]. Macrophages, T cells, and conventional dendritic cells (cDC) undergo well-characterised metabolic changes upon activation, with defined roles for glycolysis, oxidative phosphorylation (OXPHOS), and ROS in shaping immune outcomes. A recent review of the different aspects of glucose metabolism in immune cells highlighted that pro-inflammatory cells rely primarily on glycolysis – in part anaerobic –, the pentose phosphate pathway (PPP), glutaminolysis and fatty acid synthesis (FAS) while both resting and anti-inflammatory cells rather turn to OXPHOS and fatty acid oxidation (FAO) for energy supply [17]. This reflects their divergent demands for either rapid short-term or sustained and more efficient ATP production. Whether pDCs follow similar metabolic paradigms or instead rely on distinct strategies tailored to their specialised function remains unresolved.

In this review, we argue that pDC biology is fundamentally constrained and directed by redox-metabolic control mechanisms. We propose that pDCs rely on a mitochondria-centred redox programme that supports rapid IFN-I production but renders them uniquely vulnerable to metabolic collapse upon chronic stimulation. Integrating insights from redox biology, immunometabolism, and autoimmunity research, we present a novel concept that explains how pDCs transition from protective antiviral sentinels to drivers of immunopathology.

## 2 pDCs as a metabolically constrained immune cell type

Resting pDCs exhibit a distinctive cellular morphology characterised by limited cytoplasmic volume, sparse organellar content, and low biosynthetic activity [1]^,^ [18]. Compared with conventional dendritic cells (cDCs) and monocytes, pDCs display reduced phagocytic capacity and minimal engagement in antigen processing under steady-state conditions [18]. These features suggest that pDCs likely exist in a metabolically constrained state, optimised for rapid signalling rather than sustained anabolic activity. Transcriptomic analyses revealed relatively low expression of genes associated with glycolysis, lipid synthesis, and amino acid metabolism in resting pDCs [19]. Instead, pDCs appear to rely primarily on mitochondrial oxidative phosphorylation (OXPHOS) to meet basal energy demands [20]^,^ [21]. This reliance on mitochondrial metabolism may reflect an evolutionary adaptation that prioritises efficient ATP production while minimising the biosynthetic burden. Despite their low metabolic flux, pDCs arguably must maintain tight redox control even at rest. Mitochondrial respiration inevitably generates ROS, and pDCs appear to be equipped with robust antioxidant systems to prevent oxidative damage. Glutathione, thioredoxin, and peroxiredoxin pathways likely play central roles in buffering basal ROS levels, preserving cellular integrity and preventing inadvertent activation of redox-sensitive signalling pathways [22]^,^ [23]. Thus, unimpaired redox buffering mechanisms are presumably essential for maintaining immune tolerance.

## 3 Metabolic checkpoints governing pDC fate

**Fig. 1:**
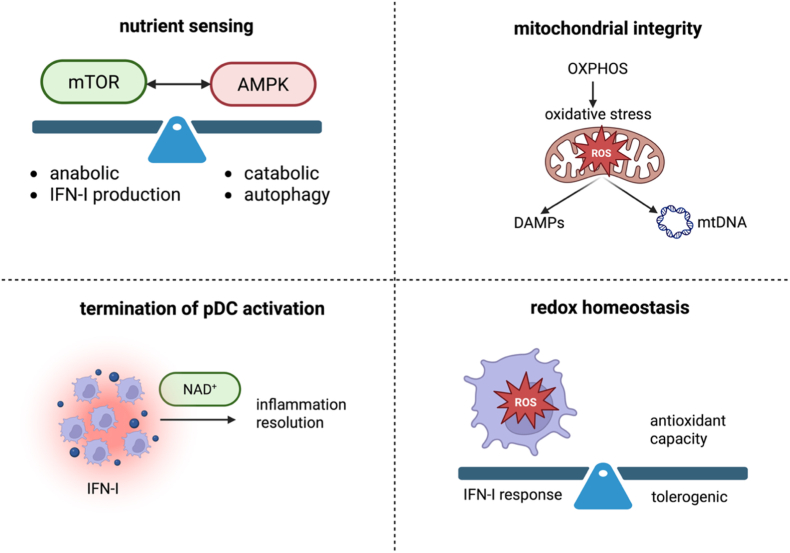
**Metabolic checkpoints of pDC activity.** Four central metabolic checkpoints likely determine whether pDCs exhibit a temporary state of activation that promptly is resolved to restore homeostasis or whether they succumb to pathogenic chronic activity. These mechanisms include a balanced activation of mTOR or AMPK in response to nutrient availability, maintenance of mitochondrial integrity despite the oxidative stress induced by OXPHOS, NAD^+^-dependent resolution of inflammation and adequate redox homeostasis achieved via a sufficient antioxidant capacity. Imbalances in these four checkpoints presumably contribute to sustained pDC activation, promoting chronic inflammation or autoimmunity.

Integrating current evidence, pDC activation can be viewed as a metabolically gated process in which distinct checkpoints determine whether an immune response is appropriately resolved or, instead, progresses towards chronic immunopathology. Rather than being passive producers of IFN-I, pDCs appear to continuously evaluate their energetic and redox state, and these parameters provide feedback to shape cell fate decisions [24]. Four central checkpoints of this regulatory network arguably include the balance between mTOR and AMPK signalling, mitochondrial fitness and dynamics, intracellular NAD^+^ availability, and the capacity of antioxidant systems to restrain ROS as illustrated in Fig. 1 [21]^,^ [25]^,^ [26].

The first of these metabolic checkpoints operates at the level of nutrient and energy sensing. mTOR is preferentially activated in nutrient-rich conditions where it promotes anabolic pathways and inhibits autophagy. Correspondingly, when nutrients are rare, mTOR activity is suppressed and catabolic pathways such as autophagy are active [27]. Engagement of TLR7/9 rapidly activates mTOR signalling, which promotes anabolic metabolism, protein translation, and the biosynthetic programmes required for high-level IFN-I production [26]. At the same time, AMPK functions as a counter-regulatory pathway that monitors cellular energy stress and limits excessive mTOR activity [28]. In physiological settings, a transient mTOR-dominant state enables rapid antiviral responses, whereas subsequent AMPK activation contributes to contraction and restoration of homeostasis. Disruption of this balance, through persistent mTOR activation or insufficient AMPK signalling, may drive pDCs towards a hyperactive phenotype incapable of resolving inflammation. A recently published study investigated the interplay between autophagy and pDC metabolism [21]. Here, the autophagy inhibitor VPS34 was shown to induce IFN-β production in the pDC cell line CAL-1 while also activating AMPK and suppressing glycolysis [21]. mTOR has been reported to be essential for TLR9-induced INF–I production in pDCs [26]. In accordance, sustained mTOR activation induced by protein kinase B (PKB) hyperactivation has been shown to elevate cytokine production by human pDCs [29]. Concordantly, in chronic hepatitis B infection marked by decreased pDC functionality, a downregulation of the PI3K-PKB-mTOR axis was observed [29]. On the other hand, mTOR inhibition by rapamycin was shown to upregulate pDC CD80 expression which led to an enhanced capacity to induce regulatory T cells and promote CD4^+^ T cell proliferation [30]^,^ [31]. This finding implies that mTOR may be a central regulator of pDC fate, determining whether cells specialise in antiviral functions or serve as linkers to adaptive immunity. However, the exact mechanisms by which rapamycin treatment in clinical practice affects the pDC compartment remain to be elucidated. Another factor determining the pDC capacity to induce adaptive immunity is mitochondrial ROS production. ROS-dependent mechanisms have recently been discovered to be indispensable for exogenous antigen cross-presentation on MHC-I by pDCs to induce CD8^+^ T cells [32]. In this regard, TLR stimulation was observed to increase mitochondrial ROS production in pDCs which enhanced their cross-presenting capacity by alkalisation of the phagosomal pH and antigen protection from degradation. However, upregulation of surface phenotypic markers of pDC maturation such as CD80/CD86 and MHC class II molecules was discovered to not depend on ROS. Interestingly, the TLR-induced ROS production was found to be independent from NADPH Oxidase 1 (NOX1) and NOX2 which utilise cytosolic NADPH to generate superoxide and have been described as indispensable for the constitutive cross-presenting capacity of cDCs. Instead, pDCs rely predominantly on mitochondrial ROS, thus limiting their ability to cross-present to metabolic states following TLR activation [32]. This highlights the relevance of not solely considering IFN-I production but also antigen presentation capacity when analysing pDC metabolism.

A second checkpoint centres on mitochondrial integrity. Because pDCs arguably rely heavily on OXPHOS to sustain IFN-I secretion, mitochondrial fitness becomes a critical determinant of functional outcome. Efficient mitophagy, balanced fission-fusion dynamics, and appropriate biogenesis ensure that damaged organelles are removed and replaced. When these quality-control mechanisms operate effectively, ROS production remains within a signalling range that supports activation without causing toxicity [33]. Conversely, accumulation of dysfunctional mitochondria leads to excessive ROS, particularly superoxide and H_2_O_2,_ release of mitochondrial DAMPs, and progressive loss of bioenergetic flexibility, all features increasingly associated with pathogenic pDCs in autoimmune disease [34], [35].

NAD^+^ metabolism represents a third checkpoint. Adequate NAD^+^ pools are required for sirtuin activity, DNA repair, and redox buffering, all of which contribute to termination of inflammatory programmes. Restoration of NAD^+^ levels following acute activation may therefore function as a molecular “reset switch,” enabling pDCs to exit the IFN-I-producing state and return to quiescence. Chronic inflammation, by contrast, drives persistent consumption of NAD^+^ through PARPs, CD38, and other enzymes, eroding this checkpoint and locking cells into a metabolically compromised, proinflammatory configuration [36]. Fourthly, the ability to manage oxidative stress likely constitutes a decisive checkpoint for pDC survival and function. Antioxidant systems, including glutathione, thioredoxin, and NADPH-dependent pathways, normally constrain ROS generated during TLR signalling and mitochondrial respiration [23]. When antioxidant defences are sufficient, ROS act as transient second messengers that help finetune IFN-I responses. When these systems are overwhelmed, however, oxidative damage amplifies inflammatory signalling, possibly perturbs endolysosomal function, and favours sustained cytokine production [34]^,^ [37]. Precisely, upon mitochondrial stress, oxidised mitochondrial DNA (Ox-mtDNA) exits into the cytoplasm where it directly activates the NLRP3 inflammasome, promoting IL-1β signalling which, in turn, further enhances the release of Ox-mtDNA [34]. Besides upregulating IL-1β, ROS-induced cellular stress has been described to inhibit IL-1 receptor antagonist (IL-1Ra) which perpetuates a pro-inflammatory phenotype [37]. While in macrophages, mitochondrial ROS have been shown to induce lysosomal dysfunction, this mechanism remains to be confirmed in pDCs [38]^,^ [39]. Thus, redox homeostasis effectively determines whether ROS serve as physiological regulators or pathological drivers.

During acute viral infection, these four checkpoints operate in a coordinated and self-limiting manner. Temporary mTOR-driven anabolic pathway amplification, controlled mitochondrial ROS generation, and timely restoration of NAD^+^ and antioxidant balance together permit a powerful, yet transient IFN-I burst, followed by resolution and return to a resting state. In contrast, chronic or repetitive stimulation, as encountered in systemic autoimmunity, presumably progressively overwhelm each of these safeguards. Persistent mTOR activity, mitochondrial dysfunction, NAD^+^ depletion, and impaired redox buffering may converge to produce a state of metabolic exhaustion in which pDCs remain trapped in a pathogenic activation loop. This checkpoint model provides a novel concept to explain the dual roles of pDCs in host defence and autoimmunity. It suggests novel therapeutic strategies aimed at restoring specific metabolic nodes, rebalancing mTOR/AMPK signalling, enhancing mitophagy, replenishing NAD^+^ pools, or reinforcing antioxidant capacity which could reprogramme pDCs from chronic IFN-I producers back towards a tolerogenic phenotype.

## 4 Endolysosomes as metabolic compartments help to control self-versus non-self-discrimination

A defining feature of pDCs is their reliance on the endolysosomal compartment for nucleic acid sensing. Endosomal maturation, acidification, and trafficking are energy-dependent processes that require coordinated metabolic input. Vacuolar ATPases consume ATP to maintain an acidic pH, while vesicular trafficking depends on cytoskeletal dynamics and membrane remodelling, demonstrating an intimate link between redox processes and endolysosomal function [40]^,^ [41]. NADPH oxidases localised to endosomal membranes generate ROS, particularly superoxide, that can modulate receptor signalling and ligand processing. In pDCs, mtROS were discovered to downregulate TLR9-induced pro-inflammatory cytokine production by blocking IRF7 in pDCs [42]. Similarly, NOX2-derived endolysosomal ROS may also contribute to fine-tuning of TLR7/9 activation thresholds, influencing the magnitude and duration of IFN-I responses. This remains to be elucidated in further studies.

The ability of pDCs to discriminate between viral and self-derived nucleic acids is central to immune tolerance. Under homeostatic conditions, degradation of endogenous RNA and DNA within properly acidified endolysosomes limits their access to TLRs. However, perturbations in metabolic or redox control may erode this protective barrier. Reduced ATP availability, impaired v-ATPase function, or dysregulated NADPH oxidase activity can alter endosomal pH and ROS levels, lowering the activation threshold for TLR engagement [43]^,^ [44]. In more detail, NOX2 is required for the translocation of TLR7 and TLR9 to the endosomal compartment while, on the other hand, NOX-deficient B lymphoblasts were described as hyper-responsive to TLR7/9 activation [44]^,^ [45]. While these studies were not performed in pDCs, it is likely that NADPH oxidase activity similarly regulates TLR signalling in pDCs. The above-described changes may allow self-nucleic acids, particularly when complexed with autoantibodies or antimicrobial peptides, to persist in signalling-competent compartments. In autoimmune settings, these vulnerabilities are likely amplified. Continuous uptake of immune complexes containing self-DNA or -RNA drives repetitive endosomal trafficking and sustained ROS generation, placing chronic demand on the redox buffering capacity of pDCs [46]. Over time, exhaustion of antioxidant systems and disruption of endolysosomal homeostasis may convert compartments that normally enforce tolerance into sites of aberrant activation. The result would be a state in which metabolic stress and defective redox regulation facilitate persistent TLR7/9 signalling, setting the stage for the chronic IFN-I production characteristic of various systemic autoimmune diseases.

## 5 Metabolic reprogramming following viral sensing

Upon engagement of TLR7 or TLR9 by viral RNA or DNA, pDCs initiate a transcriptional programme that culminates in the production of vast quantities of IFN-I [1]^,^ [3]. This process requires substantial ATP investment for chromatin remodelling, transcription, translation, and cytokine secretion. Unlike macrophages or T cells, which typically undergo pronounced glycolytic reprogramming upon activation, pDCs arguably exhibit a more restrained metabolic response[47]^,^ [48].

In the initial phase of infection with single-stranded RNA (ssRNA) viruses such as influenza and rhinovirus, as well as upon TLR7 stimulation by gardiquimod, human pDCs were observed to upregulate glycolysis but not OXPHOS [49]. After 48 h exposure to these viruses, extracellular lactate was found to be significantly increased in the supernatant which indicates that in the acute phase of viral infection, pDCs require massive and rapid energy supply in order to produce antiviral mediators such as IFN-I. Accordingly, inhibition of glycolysis by 2-deoxyglucose (2-DG) has been shown to disrupt both IFN-I production and surface expression of HLA-DR, CD80 and CD86 [49]. This indicates that in pDCs glycolysis is essential for acute IFN-I signalling and recruitment of adaptive immune cells. Importantly, neither exposure to IFN-α, nor interferon-α/β receptor (IFNAR) blockade had an impact on the virus-induced glycolysis whereas chloroquine-induced disruption of endosomal acidification inhibited this metabolic shift [49]. This strongly suggests that TLR7 signalling rather than IFNAR-mediated feedback loops is responsible for the metabolic reprogramming of pDCs during acute viral infection. This study used glycolysis inhibition, lactate production, ECAR and OCR to assess metabolic changes in pDCs. Opposingly, another study reported that IFNAR-signalling promotes OXPHOS and FAO in pDCs [50]. Moreover, this study elucidated that besides IFN-I stimulation, 24 h CpG-A-TLR9 stimulation also enhances these two metabolic pathways in pDCs. However, since this study which relied on Seahorse assay, OCR, ATP and mitochondrial membrane potential measurements utilised bone marrow-derived murine pDCs rather than PBMC-derived human pDCs, the translational relevance of these observations remains unclear and requires further investigation.

In partial contradiction with the above described findings, another study which measured OCR and mitochondrial membrane potential reported that upon acute influenza A or HSV stimulation, OXPHOS but not glycolysis was elevated in human pDCs [51]. In addition, inhibition of the electron transport chain was shown to downregulate IFN-I production within 6 h of viral stimulation. Instead of the mechanisms suggested by previous studies, this group described that AMP kinase (AMPK) was essential for HSV- and CpG-A-TLR9-induced INF–I production [51]. Since AMPK is an established driver of OXPHOS and a negative regulator of aerobic glycolysis, this supports their claim that OXPHOS, rather than glycolysis, is the central source of ATP within the acute phase of viral infection. Concordantly, another study recently reported that AMPK inhibition downregulates IFN-β production in the pDC cell line CAL-1.^21^ Another study investigated the effect of 6 h stimulation with the TLR7/8 ligand pRNA on pDC metabolism using Seahorse assay, OCR measurements and total RNA sequencing [52]. Here, OXPHOS-related gene expression was described as upregulated upon pRNA stimulation of human pDCs. Of note, pDCs were sorted by positive selection and cultured with IL-3 which may contribute to different outcomes between this and other related studies. In addition, glutaminolysis was found to be enhanced which could generate key metabolites required in the TCA cycle which subsequently fuels OXPHOS by supplying NADH and FADH_2_ [52]. However, another more recent study disagreed with the importance of glutaminolysis in TLR-stimulated pDCs despite employing a similar experimental approach [51].

**Fig. 2:**
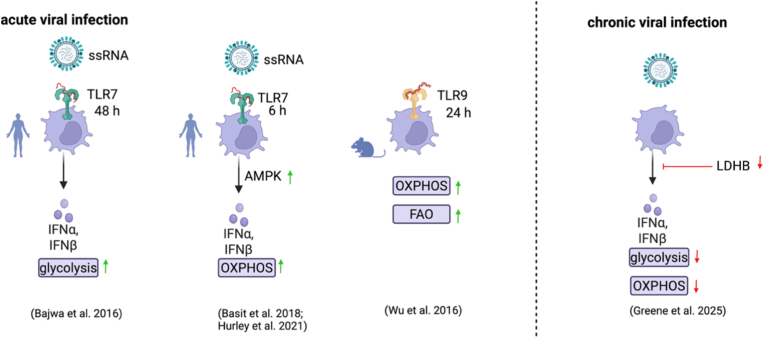
**Divergent reports on predominant metabolic pathways upon viral infection.** While most immune cells generally upregulate glycolysis to rapidly meet the increased energy demands imposed by acute viral infection, it is unclear to what extent this applies to pDCs. This figure illustrates the partly conflicting results of recent studies measuring the metabolic pathways predominantly employed by TLR-activated pDCs to enable prompt and massive IFN-I release. Differences between these outcomes may be attributed to different specimens, species, TLR ligands and duration of stimulation. Nevertheless, to unanimously clarify the exact regulation of pDC metabolism upon infection, further studies are required. During chronic viral infection, LDHB downregulation was observed to reduce both glycolysis and OXPHOS, thus limiting IFN-I production.

The discrepancy between studies reporting TLR7-induced glycolysis and those describing a predominant role of OXPHOS and FAO partly reflects the heterogeneity of experimental approaches and readouts employed to assess cellular metabolism. This is summarized in Fig. 2. Attempting to reconcile these divergent findings, one may assume that TLR7 stimulation rapidly induces glycolysis whereas IFNAR-dependent signalling could promote OXPHOS and FAO which may thus be more relevant in later phases of infection to allow for sustained energy-consuming IFN-I production. While inconsistencies between these studies may partly be attributed to differences in stimulation duration and TLR ligands, they nevertheless demand for further systematic analysis of different time points post infection to disentangle the metabolic reprogramming of pDCs upon viral infection.

A few days after the beginning of acute infection as well as during chronic infection, the IFN-I production capacity of pDCs becomes drastically reduced, reflecting a state of exhaustion [53]. A recent study identified that viral infections downregulate oxidative and glycolytic metabolism in both mouse and human pDCs via downregulation of lactate dehydrogenase B (LDHB) to limit IFN-I production [24]. This immunosuppression serves to restrict immunopathology and restore homeostasis but also renders the host more prone to secondary infections or cancer. Concordantly, LDHB expression and IFN-α production were found to be significantly decreased in pDCs derived from people with HIV (pwHIV) compared to healthy blood donors [24]. However, this experiment remains be repeated in a larger cohort to confirm these intriguing findings. Besides glucose metabolism, glycosphingolipid metabolism has recently been highlighted as an important driver of IFN-I production upon TLR9 stimulation [54]. Uridine diphosphate glucose (UDP-glucose), a substrate for UDP-glucose ceramide glucosyltransferase (UGCG) was shown to be critical for IFN-I production. Supposedly, this pathway is critical for lipid raft formation to enable IFN-I signalling. Additionally, UDP-glucose can act as an extracellular signalling molecule, binding to P2Y_14_, where it may also contribute to the inhibition of IFN-I production [55]. The multifaceted immunoregulatory role of P2Y receptors was recently reviewed by us [56].

Mitochondria in pDCs serve not only as powerhouses but also as critical signalling platforms. Activation-induced changes in mitochondrial membrane potential and electron transport chain activity lead to increased production of mitochondrial ROS (mtROS), namely superoxide and H_2_O_2_. Rather than acting solely as damaging by-products, mtROS serve as signalling molecules that amplify antiviral pathways [42]. mtROS can modulate the activity of redox-sensitive kinases, phosphatases, and transcription factors, thereby enhancing IRF7 activation and IFN-I transcription. In this context, mitochondria act as integrators of metabolic state and innate immune signalling, translating energetic flux into functional output. The tight coupling between mitochondrial activity and IFN-I production suggests that pDCs operate within a distinct redox window. Insufficient mtROS production may impair antiviral responses, whereas excessive ROS can lead to oxidative damage and dysregulated signalling that may contribute to the development of inflammatory and autoimmune diseases.

## 6 Metabolic stress during chronic activation paves the path to autoimmunity

In IFN-I-driven autoimmune diseases, pDCs are subjected to continuous stimulation, often within inflamed tissues [57]. Unlike the transient activation that occurs during acute antiviral responses, this sustained engagement of TLR7 and TLR9 pathways forces pDCs into a prolonged state of high secretory and biosynthetic demand. Maintenance of IFN-I production requires extensive transcriptional activity, protein synthesis, and vesicular trafficking, all of which impose a substantial energetic burden [13]. Consequently, chronically activated pDCs undergo pronounced metabolic reprogramming characterised by increased mitochondrial respiration and a shift towards OXPHOS to support their functional output [20]. While enhanced mitochondrial activity can initially sustain IFN-I production, prolonged reliance on this pathway is accompanied by elevated generation of ROS and progressive mitochondrial damage [21]. Excessive ROS not only compromise mitochondrial integrity but also act as secondary signalling mediators that can further potentiate inflammatory pathways, including NF-κB and IRF signalling [58]. Under physiological conditions, mitochondrial quality control mechanisms, such as mitophagy and mitochondrial biogenesis, serve to limit this damage. However, accumulating evidence suggests that chronic inflammatory environments impair these homeostatic processes [21]^,^ [25]^,^ [59]. Defective mitophagy allows dysfunctional mitochondria to accumulate within pDCs, perpetuating oxidative stress and promoting the release of mitochondrial DNA and other DAMPs that can reinforce TLR activation [60]. This arguably establishes a self-amplifying feed-forward loop in which metabolic dysfunction and innate immune signalling continuously drive one another. In this regard, the release of Ox-mtDNA has recently been proposed as a central determinant of pDC-driven autoimmunity [34]. Ox-mtDNA has repeatedly been shown to induce IFN-I production in pDCs, reflecting their major function in innate immunity [61]^,^ [62]. However, recently, Ox-mtDNA sensing by TLR9 in pDCs was revealed to induce NLRP3-inflammasome-IL-1β signalling, essential for T_FH_ cell differentiation and autoantibody production [34]. This provides novel insights into the role of pDCs as key intermediaries linking innate and adaptive immunity and may transform our understanding of how persistent inflammation can progress to overt autoimmune disease. Interestingly, the pDC Ox-mtDNA-NLRP3-IL-1β-T_FH_ axis may represent a previously overlooked therapeutic target, suggesting that IL-1 or IL-1R antibodies could be employed to treat autoimmune diseases by possibly limiting autoantibody production. This potential mechanism remains to be further explored in future studies.

An additional layer of metabolic vulnerability in chronically activated pDCs presumably involves disruption of cellular NAD^+^ homeostasis. Chronic inflammation is associated with increased activity of NAD^+^-consuming enzymes such as poly(ADP-ribose) polymerases (PARPs), CD38, and sirtuins, all of which are induced by DNA damage and inflammatory signalling [36]^,^ [63]. Sustained activation of these pathways can progressively deplete intracellular NAD^+^ pools, compromising multiple metabolic processes. In pDCs, reduced NAD^+^ availability is likely to impair the activity of mitochondrial sirtuins (e.g., SIRT3 and SIRT5), which are essential for maintaining efficient oxidative phosphorylation, regulating antioxidant defences, and promoting mitophagy [25]. Loss of sirtuin-mediated control may therefore exacerbate mitochondrial dysfunction, increase ROS accumulation, and diminish the capacity of pDCs to restore metabolic balance after activation. NAD^+^ depletion may also have direct consequences for the epigenetic and transcriptional programmes that determine pDC identity. Sirtuin-dependent deacetylation of histones and transcription factors contributes to the resolution of inflammatory responses and the termination of IFN-I signalling [64]. Insufficient NAD^+^ could thus “lock” pDCs into a pathogenic state by preventing the normal shutdown of IRF7-driven gene expression. Together, impaired mitochondrial quality control and NAD^+^ deficiency likely create a metabolic landscape that favours persistent activation rather than the return to quiescence. In this context, pDCs arguably become trapped in a maladaptive programme characterised by sustained IFN-I secretion, chronic oxidative stress, and reduced capacity for immunological tolerance. These interconnected processes suggest that metabolic exhaustion is not merely a consequence of chronic pDC activation but a central driver of their pathogenic behaviour in autoimmunity. Targeting pathways that restore mitochondrial fitness or replenish NAD^+^ levels may therefore represent promising strategies to break the cycle of metabolic stress and aberrant IFN-I production in autoimmune disease [63].

## 7 pDC metabolism in autoimmune diseases

pDCs play a central role in the pathogenesis of autoimmune diseases with an elevated IFN-I signature such as systemic sclerosis (SSc) [65]. Increased pDC numbers in the skin and lungs, accompanied by reduced rates of circulatory pDCs have been described in SSc patients. While patients with the milder limited cutaneous form of Ssc (lcSSc) often present with anti-centromere antibodies (ACA), the more severe diffuse cutaneous SSc (dcSSc) is frequently associated with anti-topoisomerase I antibodies (anti-Scl-70; ATA) [66]. Interestingly, pDCs from ACA^+^ patients demonstrated higher dependence on OXPHOS and decreased glycolytic capacity compared to pDCs from ATA^+^ patients [20]. This implies, that in more severe dsSSc, pDCs rely less on OXPHOS but rather on glycolysis for energy supply.

Orchestrating an immune response upon TLR activation causes extensive demand for protein folding, modification and secretion which can lead to accumulation of misfolded proteins in the ER and consequently to the activation of the unfolded protein response (UPR) [67]. An important sensor of such ER stress, inositol-requiring enzyme 1α (IRE1α), enables activation of the transcription factor X-box binding protein 1 (XBP1) which can modulate immune cell metabolism in various manners. It has recently been reported that IRE1α-XBP1 decreases TLR9-induced IFN-I production in pDCs by inducing phosphoglycerate dehydrogenase (PHGDH), thus inhibiting the intracellular ATP-cAMP-CREB axis [47]. Precisely, PHGDH was observed to shift glycolysis away from pyruvate production, thereby reducing ATP synthesis via the TCA cycle and electron transport chain route. Interestingly, PHGDH expression was shown to be strongly downregulated in chronically activated pDCs from patients with either SSc or SLE. This effect was supposedly mediated by chemokine (C-X-C motif) ligand 4 (CXCL4), which is present at high levels in the context of SSc. Accordingly, the small-molecule TCA cycle disruptor CPI-613 reduced TLR9-driven IFN-I production in healthy pDCs in vitro [68]. Testing this drug in the context of chronically activated pDCs of SSc or SLE patients could add to our understanding of the metabolic pathways dictating disease pathogenesis and progression.

**Fig. 3:**
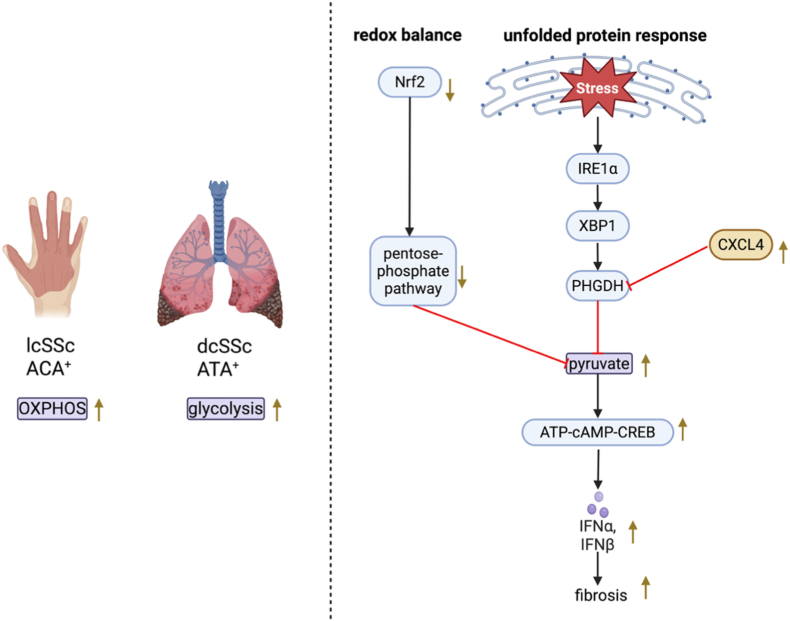
**Metabolic dysregulation in SSc pathogenesis**. While in the limited cutaneous form of SSc, OXPHOS was discovered as the predominant metabolic pathway, glycolysis was found to be upregulated in the more severe diffuse cutaneous Ssc. On the right, two metabolic pathways which are dysregulated in SSc are depicted. First, expression of NRF2 is reduced, leading to a decreased shift towards the pentose phosphate pathway, thus causing redox imbalance in terms of lower antioxidant capacity. In addition, since more glucose is hence available for pyruvate production, the ATP-cAMP-CREB axis is promoted, leading to enhanced IFN-I production by pDCs and worsened clinical outcomes. Second, the chemokine CXCL4 is upregulated in SSc which inhibits PHGDH, thus disrupting the unfolded protein response by increasing pyruvate levels and consequently INF–I production.

Another mechanism which diverts glucose metabolism away from pyruvate production is the stress-activated nuclear factor erythroid 2-related factor 2 (Nrf2)-mediated induction of the pentose phosphate pathway (PPP) which simultaneously plays a major role in maintaining redox balance [69]. Precisely, Nrf2 was found to directly upregulate glucose-6-phosphate dehydrogenase (G6PD), the rate-limiting enzyme of the PPP [69]. Thereby, NADPH production is likely enhanced which allows for regeneration of glutathione, an important cellular antioxidant [70]. Simultaneously, the lack of pyruvate reduces intracellular ATP levels and thus impedes pDC activation. This process is triggered by skin stiffness and serves to restraint pDC activation during wound healing. Of note, this pathway has been shown to be dysregulated in the skin of SSc patients, thus promoting chronic pDC activation and fibrosis [69]. This is an example of insufficient regulation of pDC metabolism leading to persistent activity and immunopathology (see Fig. 3). Concordantly, in other immune cell types, Nrf2 has repeatedly been described as an important anti-inflammatory mediator [71]^,^ [72]. For instance, Nrf2-deficient BMDCs exhibited upregulated expression of co-stimulatory molecules and more strongly induced pro-inflammatory T cell differentiation [73]. In addition, Nrf2-deficiency was observed to increase ROS levels in murine immature DCs [74]. However, the mechanism by which Nrf2-deficiency promotes this pro-inflammatory DC phenotype has been argued to be independent from ROS and rather reliant on the p38-MAPK-CREB/ATF1 axis [75]. In macrophages, Nrf2 has been shown to be stabilised upon sensing of high ROS levels by Mst1/2 to serve as a cell-intrinsic protection mechanism against oxidative damage [76]. While this has not yet been examined, it is likely that Nrf2 exerts similar functions in pDCs.

In the context of SLE, pDCs have been described as functionally impaired which has recently sparked uncertainty about their relevance in SLE pathogenesis [77]. On the contrary, recent clinical trials have shown promising results in the avenue of depleting pDCs in SLE and CLE by the means of monoclonal antibodies directed against BDCA2 and ILT7 [78]^,^ [79]. It would thus be interesting to evaluate potential metabolic pathway alterations in pDCs of SLE patients to further understand their role in this disease. Due to oxidative stress and reduced energy generation in SLE, patient sera contained lower amounts of amino acids and organic acids while fatty acids were found to be increased [80]^,^ [81]. Interestingly, expression of the amino acid transporters *SLC3A2*, *SLC7A5* and *SLC7A11* was found to be elevated in pDCs from kidney biopsies of lupus nephritis patients, supposedly increasing mTORC1 activation and thus reprogramming pDC metabolism. Since this axis was found to be dependent on JAK2-STAT5 signalling, the authors suggest JAK2 inhibitors, which are already widely used in treatment of other rheumatic diseases, as potential metabolic regulators of SLE disease activity [82]. A deepened understanding of physiological and pathological pDC metabolic regulation could hence contribute to better tailored therapeutic approaches in autoimmune diseases.

## 8 Therapeutic implications: targeting metabolic vulnerabilities of pDCs

Recognising that pDC activation is tightly coupled to cellular metabolism and redox biology opens new avenues for therapeutic intervention in IFN-I-driven autoimmune diseases. Traditional approaches have focused largely on blocking upstream receptors or neutralising IFN-I after production [83]. The emerging view of pDCs as metabolically regulated cells suggests an additional strategy: rather than directly suppressing immune signalling, it may be possible to reprogramme the metabolic state that sustains pathological activation. Targeting metabolic and redox checkpoints offers the potential to selectively dampen chronic IFN-I production while preserving the capacity for protective antiviral responses. One attractive node arguably is the mTOR–AMPK axis. Hyperactivation of mTOR signalling has been implicated in several autoimmune disorders and is known to promote anabolic programs required for sustained cytokine production [84]. Pharmacologic mTOR inhibitors such as rapamycin (sirolimus) already show clinical benefit in SLE, and part of their efficacy may be attributed to effects on pDC function [85]. Conversely, agents that activate AMPK, such as AICAR or metformin, could favour a more quiescent metabolic profile, limiting excessive pDC activation and promoting resolution of inflammation. For instance, the conventional synthetic disease modifying anti-rheumatic drugs (csDMARD) methotrexate and sulfasalazine promote AICAR accumulation [86]. While this increases the release of anti-inflammatory adenosine in the treatment of rheumatoid arthritis, AICAR-induced AMPK activation may additionally shape an anti-inflammatory metabolic immune cell profile. Similarly, the anti-inflammatory function of metformin can in part be explained by AMPK induction and thus reprogramming of immune cell metabolism [87]^,^ [88]. Therefore, modulation of the mTOR-AMPK balance represents a rational approach to restore physiological control over IFN-I output without complete immunosuppression.

A second possible therapeutic avenue involves restoration of mitochondrial quality control. Enhancing mitophagy or supporting mitochondrial biogenesis may interrupt the cycle of ROS accumulation and inflammatory reinforcement characteristic of chronically activated pDCs. Small molecules that promote PINK1/Parkin-dependent mitophagy, stabilise mitochondrial membrane potential, or improve electron transport chain efficiency could theoretically normalise pDC bioenergetics and reduce aberrant signalling [21]^,^ [89]. Although such strategies are still largely unexplored in the context of pDC biology, they offer a means to address the root cause of metabolic exhaustion rather than its downstream consequences.

Replenishment of intracellular NAD^+^ pools may represent another promising intervention. NAD^+^ precursors such as nicotinamide riboside and nicotinamide mononucleotide have been shown in other systems to enhance mitochondrial function, reduce oxidative stress, and improve cellular resilience [90]^,^ [91]. In pDCs, restoration of NAD^+^ availability could reactivate sirtuin-dependent programmes that terminate IFN-I signalling, reinforce antioxidant defences, and promote recovery after activation. In parallel, inhibition of major NAD^+^-consuming enzymes, including PARPs or CD38, may help preserve NAD^+^ levels in chronically inflamed tissues [92]. These approaches offer a mechanistically grounded means to reset dysfunctional pDCs to a more tolerogenic state. Given the importance of redox regulation in endolysosomal TLR signalling, modulation of ROS-generating or ROS-scavenging pathways presumably also holds therapeutic potential. Selective inhibition of NADPH oxidases, reinforcement of glutathione or thioredoxin systems, or delivery of mitochondria-targeted antioxidants could recalibrate the redox environment that shapes TLR7/9 activation thresholds [33]. Such interventions might be particularly effective in settings where immune complexes and self-nucleic acids chronically stimulate pDCs, as they could raise the signalling threshold required for pathological activation without abolishing responsiveness to genuine pathogens.

**Fig. 4:**
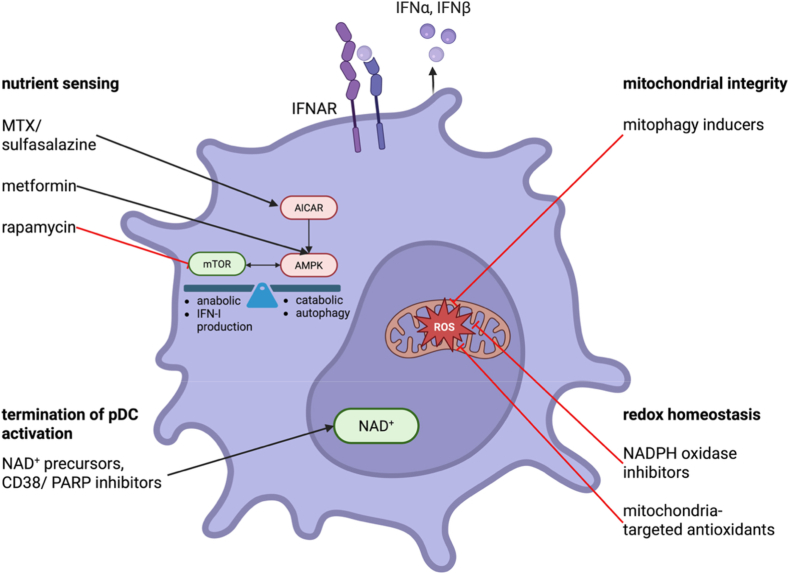
**pDC metabolism as a therapeutic target in autoimmunity and hyperinflammation.** During chronic inflammation and autoimmunity, pDC metabolism and redox homeostasis are disrupted in diverse ways. Selectively addressing these checkpoints may allow for a fine-tuned resolution of inflammation without impairing pDC functionality against pathogens. On the top left, mechanisms are depicted by which the anabolic mTOR could be inhibited and the catabolic AMPK enhanced to reduce IFN-I production. On the bottom left, methods to increase intracellular NAD^+^ levels are proposed which could benefit the resolution of inflammation. On the top right, mitophagy inducers are suggested to restore mitochondrial integrity while on the bottom right, different strategies to reduce mitochondrial ROS production are outlined to maintain redox homeostasis and terminate aberrant pDC activity.

Finally, the metabolic perspective provides a novel concept for understanding and refining emerging pDC-directed therapies (see Fig. 4). Agents that deplete pDCs, block their trafficking, or inhibit TLR7/9 signalling are already in clinical development [78]^,^ [79]. Combining these approaches with metabolic modulators may enhance efficacy or permit lower dosing by reducing the intrinsic fitness of pathogenic pDCs. Moreover, metabolic biomarkers, such as indicators of mitochondrial dysfunction or NAD^+^ depletion, could help identify patient subsets most likely to benefit from specific interventions[63]^,^ [93].

## 9 Conclusion

Collectively, these considerations suggest that metabolic reprogramming of pDCs represents a complementary strategy to conventional immunomodulation. By targeting the energetic and redox foundations that sustain chronic IFN-I production, it may be possible to convert pDCs from drivers of autoimmunity into cells capable of re-establishing immune tolerance. Future studies defining the precise metabolic requirements of human pDCs *in vivo* will be essential for translating these concepts into safe and effective therapies.

## Funding

This research did not receive any specific grant from funding agencies in the public, commercial, or non-profit sectors.

## CRediT authorship contribution statement

**Lena Rueschpler:** Investigation, Visualization, Writing – original draft. **Sebastian Schloer:** Conceptualization, Funding acquisition, Project administration, Resources, Supervision, Writing – original draft, Writing – review & editing.

## Declaration of competing interest

The authors declare no conflict of interest. The funding sponsors had no role in the design of the study; in the collection, analyses, or interpretation of data; in the writing of the manuscript, and in the decision to publish the results.

## Data Availability

No data was used for the research described in the article.
